# SGA-DT: An adaptive fusion framework for missing data imputation and interpretable healthcare classification

**DOI:** 10.1371/journal.pone.0343619

**Published:** 2026-03-26

**Authors:** Monalisa Jena, Satchidananda Dehuri, Sung-Bae Cho

**Affiliations:** 1 Department of Computer Science, Yonsei University, Seoul, South Korea; 2 Department of Computer Science, Fakir Mohan University, Balasore, Odisha, India; Dr Shakuntala Misra National Rehabilitation University, INDIA

## Abstract

Despite advances in machine learning and medical data processing, handling missing values remains a critical and complex challenge in healthcare analytics. Missing data, especially in non-class attributes can severely compromise model accuracy, clinical reliability, and interpretability. In sensitive domains such as healthcare, improper imputation may lead to biased outcomes or delayed interventions. To address this challenge, we propose SGA-DT, an adaptive and interpretable learning framework that combines the best features of genetically optimized support vector regression (SVR) with a decision tree (DT) classifier for robust healthcare prediction. The framework adaptively selects an imputation strategy based on the level of missingness. It uses standard SVR for low, iterative SVR for moderate, and k-Nearest Neighbor (KNN) followed by SVR refinement for high missingness. Genetic algorithm (GA) is used to select the best SVR kernel and tune its hyperparameters, enhancing imputation accuracy across different data patterns. The complete dataset is then classified using DT, providing both robustness and transparency in prediction. The SGA-DT framework is evaluated on three healthcare datasets, Breast Cancer, Mammographic, and Hepatitis, along with other real-world and synthetic datasets. For interpretability analysis, decision trees are generated under varying missingness levels to support clinical transparency. Comparative results show that SGA-DT consistently outperforms multiple integrated frameworks across accuracy, precision, recall, and F-measure, demonstrating its robustness, interpretability, and generalizability in healthcare prediction tasks.

## 1 Introduction

In data analysis and machine learning, handling missing data is a critical challenge that can significantly affect model performance and downstream decision-making. Missing values arise due to various reasons, such as incomplete data collection, data corruption, or transmission loss [1,2]. In the healthcare domain, missing data is especially common in patient records, where incomplete diagnostic, demographic, or sensor-based information can lead to biased analyses or incorrect clinical decisions [3,4]. Effective imputation is thus essential to preserve the integrity, reliability, and accuracy of predictive models used in such sensitive applications. In classification tasks, non-class attributes, also referred to as predictor or independent variables are particularly crucial, and missingness in these features can severely degrade classification accuracy. Traditional imputation methods, including statistical techniques such as mean, median, and mode, are widely used due to their simplicity and low computational cost [5,6]. However, these methods typically ignore the inter-variable relationships, often resulting in suboptimal imputations. Studies have demonstrated that capturing dependencies among features leads to more accurate estimations of missing values [7,8]. In this context, SVR offers a compelling solution, as it is capable of modeling complex nonlinear relationships between variables.

In this work, we leverage SVR to impute missing values in non-class attributes and integrate it with a decision tree (DT) classifier to form a unified framework. To further enhance imputation accuracy, Genetic Algorithm (GA) is employed to optimize both the SVR kernel type and its hyperparameters. In healthcare scenarios, interpretability is essential for model acceptance and trust, as clinical decisions require transparent and explainable reasoning rather than opaque model outputs [9,10]. Models lacking interpretability are often unsuitable for sensitive applications where predictions directly influence medical decisions. The proposed SGA-DT framework therefore addresses the dual challenge of missing value imputation and classification while ensuring interpretability through the DT component.

SVR is a robust machine learning technique based on the principles of Support Vector Machines (SVMs) [11,12]. It is designed to perform regression by constructing an optimal hyperplane that minimizes prediction errors [13,14]. Its capacity to model complex, nonlinear feature relationships makes it well-suited for imputing missing values in non-class attributes. Decision trees, on the other hand, are hierarchical models that split data based on feature values and produce interpretable decision rules for classification. Due to their interpretability, flexibility, and ability to handle both numerical and categorical inputs, DTs are widely adopted in predictive modeling, especially in the healthcare domain [15]. The proposed SGA-DT framework combines GA-optimized SVR for imputation with DT for classification, leveraging the strengths of each component to deliver robust, interpretable, and accurate predictions even in the presence of incomplete data.

The primary objective of integrating SGA with DT is to enhance both classification accuracy and model interpretability, particularly in the presence of missing data. The major contributions of this paper are summarized as follows:

- An integrated SGA-DT framework is proposed, where a GA-optimized SVR model imputes missing values in non-class attributes, followed by interpretable classification using a Decision Tree. This integration balances prediction accuracy and model transparency.
- An adaptive imputation strategy is designed, which dynamically selects the most suitable technique among canonical SVR, iterative SVR, or KNN with SVR refinement, based on the proportion of missing data, enhancing robustness across diverse datasets.
- Genetic Algorithm is employed to jointly optimize the SVR kernel type (Linear, RBF, Polynomial) and its hyperparameters (C,γ,ϵ,d), enabling the model to adapt effectively to heterogeneous data distributions and improving imputation accuracy.
- An automatic kernel selection mechanism is embedded within the GA search space, guided by correlation structure and dataset characteristics, thereby improving the flexibility and adaptability of the imputation process.
- The framework is generalizable across both categorical and continuous missing attributes and demonstrates strong applicability in healthcare and other real-world domains where data incompleteness and interpretability are critical concerns.
- To enhance interpretability, pruned decision trees are generated on imputed datasets under varying missingness conditions (low, medium, high). Cost-complexity pruning is applied to prevent overfitting while preserving clear, rule-based explanations essential for sensitive domains like healthcare.

The remainder of this paper is organized as follows. The Related works section reviews existing studies relevant to the present research. The Proposed work section describes the SGA-DT framework in detail, including the algorithmic formulation, schematic diagrams, and computational complexity analysis. The Experimental results section presents the dataset description, performance evaluation, and detailed discussions of the obtained results. The contribution of individual components of the proposed framework is systematically analyzed in the Ablation study section. Possible limitations and potential risks affecting the proposed framework are discussed in the Threats to validity section. The Conclusion and future work section summarizes the key findings and outlines directions for future research.

## Related work

Several studies have explored missing value imputation, focusing on different techniques and their effectiveness across various applications. In this section, significant contributions in this field are discussed, focusing on different techniques and their effectiveness across various applications. Idri et al. [16] examined the impact of missing data imputation on software development effort estimation (SDEE), introducing an SVR-based imputation technique for two analogy-based estimation methods: classical analogy and fuzzy analogy. Their study compared SVR and KNN imputation across seven datasets, three missingness mechanisms, and missing data percentages ranging from 10% to 90%. The results indicate that SVR-based imputation improves predictive accuracy over KNN and reduces the negative impact of missing data on estimation performance. Additionally, fuzzy analogy consistently outperformed classical analogy in terms of standardized accuracy, regardless of the imputation method or dataset characteristics. Table 1 summarizes the key contributions in this field, highlighting the major studies, methodologies, and findings related to missing data imputation.

**Table 1:** Comparison of existing frameworks integrating SVR with other machine learning models for missing value imputation in non-class attributes.

Author(s)	Year	Imputation Technique	Imputation technique(s) Compared with /Analyzed	Performance Metrics	Application Area	Dataset Details
Aydilek and Arslan [19]	2013	Hybridization of fuzzy c-means, SVR and a genetic algorithm.	Fuzzy c-means genetic algorithm (GA) imputation, SVR genetic algorithm imputation, and zero imputation	Root mean squared error (RMSE), accuracy	Machine learning and data mining	Six datasets from UCI machine learning repository
Idri et al. [16]	2018	SVR	k-nearest neighbor (KNN) imputation	Median standardized accuracy (SA)	Software development effort estimation	Albrecht, COCOMO81, China, Desharnais, Kemerer, and Miyazaki datasets from the PRedictOr Models In Software Engineering (PROMISE) data repository and The International Software Benchmarking Standards Group data repository (ISBSG)
Shang et al. [17]	2018	Hybridization of fuzzy c-means, particle swarm optimization and SVR	GA and fuzzy c-means (GA-FCM), KNN and non-Parametric Regression (KNN-NPR), PSO-SVR	RMSE and Relative Accuracy (RA).	Transportation analytics.	Traffic volume dataset from the urban expressway and urban arterial road
S. M. Mostafa [20]	2019	Cumulative linear regression	Different R and python packages used for missing value imputation	Imputation time, RMSE, MAE, and Coefficient of Determination (CoD)	Handling missing data in statistical analysis and machine learning models	Diabetes, graduate admissions, profit estimation of companies, red & white wine dataset, California diamonds datasets from several sources
Raja and Thangavel [21]	2020	Rough K-means centroid-based imputation	k-means centroid-based imputation, fuzzy C-means centroid-based imputation, k-means parameter-based imputation, C-means parameter-based imputation, and rough k-means parameter-based imputation methods	RMSE and MAE	Data analysis in healthcare and biological research domains	Dermatology, Pima, Wisconsin, and Yeast datasets from UCI repository
M.A. H. et al. [18]	2022	SVR	ANN, KNN, and General regression neural network (GRNN)	MSE, MAE, MAPE, and computational time	Ground electromagnetism in space weather applications	MAGDAS-9 ground electromagnetism data
A. McDonald [22]	2022	Imputation using empirical relationships or machine learning models from offset wells.	Removal of depth levels with missing values	RMSE and MAE	Handling missing data in petrophysical well log datasets.	Well log data from the oil and gas industry.
Kurniadi et al. [23]	2023	Local Mean Imputation (considering class uniqueness)	Global mean imputation, Deletion of records	Accuracy, precision, recall	Rock facies classification	The well logging data published by the University of Kansas
Xu et al. [24]	2024	SVR based on the finite element method and time input	PSO Optimization for SVR	Accuracy, computational time	Earth-rockfill dam settlement monitoring	Real-world earth-rockfill dam monitoring data
Punitha and Sathiaseelan [25]	2024	Ensemble Regression Model (ERM) using SVR	ERM using SVR, MLP, and LR	Accuracy, precision, recall, F-measure	Detection of health issues in Internet of Medical Things (IoMT) applications	cStick dataset (IoMT-based medical dataset
Karimov et al. [26]	2025	SVR	Multiple Imputation by Chained Equations (MICE), KNN	Accuracy, precision, recall, F-measure	Sarcopenia diagnosis	Sarcopenia research dataset

S. M. Mostafa [17] introduced a cumulative linear regression-based imputation algorithm, which iteratively incorporates previously imputed variables into the regression model to improve missing data estimation. The proposed method was compared with existing Python and R packages using five datasets with varying missingness mechanisms, and performance was evaluated based on imputation time, root mean square error (RMSE), mean absolute error (MAE), and coefficient of determination (*R*^2^). The findings indicate that the proposed algorithm slightly outperforms existing methods, with performance variations depending on dataset size, missing percentage, and missingness type. M.A. H. et al. [18] investigated missing data imputation in space weather applications, focusing on ground electromagnetism data from the MAGDAS-9 dataset. They evaluated artificial neural networks (ANN), KNN, SVR, and general regression neural network (GRNN) for imputing missing values, comparing them against traditional statistical methods such as zero-value substitution, listwise deletion, mean substitution, and hot deck imputation. Their results showed that SVR achieved the best performance, reducing reconstruction errors with an MSE of 0.314 and MAPE of 0.738, improving imputation accuracy by up to 80%. Their study highlights the significance of machine learning-based imputation in enhancing space weather event characterization and analysis.

Xu et al. [24] addressed the challenge of missing data in earth-rockfill dam settlement monitoring by proposing an imputation model based on SVR integrated with the finite element method (FEMTSVR). To enhance performance, they developed an improved particle swarm optimization (IPSO) algorithm for hyperparameter tuning. Additionally, a sequential prediction model using gate recurrent unit (GRU) networks was introduced to monitor dam behavior on both imputed and complete datasets. Their evaluation on real-world dam monitoring data demonstrated high efficiency in handling large-scale missingness, providing a robust model for structural health monitoring in civil and hydraulic engineering. Karimov et al. [21] explored the impact of missing data in sarcopenia research and evaluated multiple imputation by chained equations (MICE), SVR, and KNN as imputation techniques. Their findings indicate that gradient boosting consistently achieved the highest classification accuracy, while KNN and MICE effectively preserved data integrity, improving predictive performance. Their study underscores the importance of choosing appropriate imputation methods to enhance the reliability of medical datasets.

From a comprehensive analysis of related works in this field, it has been observed that SVR is increasingly favored for missing data imputation, as it outperforms traditional methods in both accuracy and robustness. Studies indicate that SVR-based imputation effectively maintains data distribution and minimizes error rates (RMSE, MAE), making it suitable for complex datasets across various domains. Furthermore, hybrid approaches that integrate SVR with clustering techniques (e.g., Rough K-means, FCM) and optimization algorithms (e.g., PSO, GA) have been shown to enhance imputation performance. The findings reinforce SVR’s effectiveness across different missingness levels, establishing it as a key machine learning-driven approach for improving data reliability and predictive accuracy.

However, despite these strengths, most studies lack an adaptive mechanism that selects imputation strategies by missingness level and attribute type. They also rarely optimize SVR kernel selection and hyperparameters jointly, limiting generalizability across heterogeneous healthcare data. In addition, interpretability is often overlooked, hindering clinical trust. The proposed SGA-DT framework addresses these gaps via GA-optimized, kernel-aware SVR with adaptive imputation (low/medium/high missingness) and an interpretable Decision Tree classifier.

To explicitly connect the gaps identified in the literature with the goals of this work, Table 2 summarizes the main limitations of existing imputation methods, including traditional ML and recent deep learning based approaches, and highlights how the proposed SGA-DT framework addresses each of these gaps.

**Table 2:** Key contributions of the proposed SGA-DT framework in addressing identified research gaps.

Identified gap in literature	Limitations in existing ML / deep learning approaches	Contribution of SGA-DT
Lack of adaptive imputation based on missingness levels	Classical SVR/KNN treat all missingness levels uniformly, while deep models require large datasets and degrade under high missingness in small healthcare samples.	Adaptive imputation using thresholds $\left(\tau_{1},\tau_{2}\right)$ to handle low, medium, and high missingness more robustly
No joint optimization of SVR kernel and hyperparameters	Most ML imputers fix kernel choice and tune few parameters; deep learning based imputation models (deep imputers) rely on backprop but still require extensive data to generalize	GA-based joint optimization of kernel type and (C,ϵ,γ) for robust imputation across heterogeneous attributes
Limited interpretability, critical in healthcare	Deep imputers (AE, VAE, GAN-based) often act as black-box latent models, offering little clinical transparency	Post-imputation Decision Tree classifier yields transparent, rule-based predictions suitable for medical decision support
Poor generalizability across datasets with different missingness patterns	Deep imputers may overfit small healthcare datasets; simple ML methods degrade under distribution shifts	Correlation-based feature filtering and GA-optimized SVR improve stability, while DT provides interpretable downstream classification

## Proposed work

The proposed SGA-DT framework integrates adaptive imputation, kernel-aware SVR optimization, and interpretable Decision Tree classification to address the challenges posed by missing values in heterogeneous healthcare datasets. This section first formalizes the problem by defining the imputation and optimization objectives, followed by a detailed description of the framework architecture and its key components.

### Problem statement

Given an input dataset *X* consisting of *n* instances and *p* attributes, where *p* = *q* + *r*, such that *q* represents the class attribute and *r* represents the non-class attributes. The dataset is divided into two subsets:

- *X*_*c*_: The subset of *X* with complete attribute values:
  $$X_{c}=\{(x_{i},y_{i})|x_{i}\in X,y_{i}\in \{c_{1},c_{2},...,c_{k}\}\}$$ (1)
  where *x*_*i*_ contains no missing values, and *k* is the number of possible class labels.
- *X*_*m*_: The subset of *X* with missing values in non-class attributes:
  $$X_{m}=\{(x_{i},y_{i})|x_{i}\in X,y_{i}\in \{c_{1},c_{2},...,c_{k}\}\}$$ (2)
  where some features in *x*_*i*_ are missing.

The objective is to impute missing values in *X*_*m*_ using an adaptive SVR model optimized by GA and then classify the imputed dataset using DT. Let *X*_*c*_′ be the subset of *X*_*c*_ with no class attributes, and *X*_*m*_′ be the subset of *X*_*m*_ with no class attributes. Let $f:X_{c}^{\prime }\to R$ be the imputation function that predicts missing values in *X*_*m*_′, where the strategy is selected adaptively based on missingness level and the SVR configuration is optimized using GA.

The imputation function *f* is dynamically selected based on the missingness percentage (λ):


$$\begin{array}{c} f(x_{i})=\left\{ \begin{array}{cc} \text{SVR}, & \text{if }\lambda \leq \tau_{1}\text{ (low missingness)}\\ \text{Iterative SVR}, & \text{if }\tau_{1}<\lambda \leq \tau_{2}\text{ (moderate missingness)}\\ \text{KNN-SVR Refinement}, & \text{if }\lambda >\tau_{2}\text{ (high missingness)} \end{array}\right. \end{array}$$ (3)

where $\tau_{1}$ and $\tau_{2}$ are predefined thresholds that classify missingness as low (<10%), moderate (10–25%), and high (>25%). We set the thresholds $\tau_{1}$ = 10% and $\tau_{2}=25\%$ by adapting cut-offs that are widely used in empirical studies on missing-data patterns [27]. Although these works are not healthcare-specific, an exploratory analysis of the missing-value percentages in our datasets showed that these thresholds naturally partition attributes into low (<10%), moderate (10–25%), and high (>25%) missingness groups. Consequently, $\tau_{1}$ and $\tau_{2}$ serve as reasonable operating points for defining the three adaptive imputation regimes used in this work. Missingness below 10% is often considered negligible and suitable for simple imputation methods like SVR. If the missingness is between 10–25%, then missing values start affecting model stability, which requires more robust techniques such as Iterative SVR. Beyond 25% of missingness, imputation becomes increasingly error-prone, and combining global modeling with local refinements (e.g., KNN-SVR) helps maintain data fidelity. The set of imputed missing values is defined as:


$$\hat{X_{m}}=\{(x_{i},y_{i},f(x_{i}))|(x_{i},y_{i})\in X_{m}^{\prime }\}$$ (4)

where, *f*(*x*_*i*_) represents the predicted values obtained from the GA-optimized SVR (SGA) model. The dataset after imputation is:


$$X^{\prime }=X_{c}+\hat{X_{m}}$$ (5)

Here *X*′ contains the complete dataset with imputed missing values. A decision tree classifier *T* is trained on *X*′, and the predicted class label for an instance *x*_*i*_ is:


$${\hat{y}}_{x_{i}}=T(x_{i})$$ (6)

### Optimization objective

The objective is to find suitable hyperparameter configurations for the SVR model $\hat{f}$ and the decision tree *T* that maximize classification accuracy while ensuring that SVR-imputed values remain within an acceptable error margin:


$$\min\frac{1}{n}\sum_{i=1}^{n}[\hat{y}(x_{i})\neq y_{i}]\;\text{s.t.}\;\|\hat{f}(x_{i})-x_{i}\|\leq \varepsilon ,\;\forall (x_{i},y_{i})\in X_{m}^{\prime }$$ (7)

where ε is the user-defined prediction error threshold ensuring imputed values remain within an acceptable deviation from actual values, ‖·‖ denotes the Euclidean norm to measure imputation accuracy, and ε>0 is a predefined tolerance value selected within the range [0.001, 0.1] based on empirical validation, ensuring a balance between imputation precision and model generalization. In this work, ε is selected from the interval [0.001, 0.1] based on preliminary experiments that examined the trade-off between imputation precision and model stability. Once chosen, this value remains fixed throughout the optimization process. [·] is the indicator function that returns 1 if the condition is true, and 0 otherwise. The objective function aims to minimize the classification error rate, while ensuring high-quality imputation from the SVR model.

Since hyperparameter optimization can increase the risk of overfitting, preventive measures are introduced in the evaluation procedure. Hyperparameter settings are assessed using 5-fold cross-validation on the training data, and generalization performance is reported on a separate held-out test set. Residual risks of overfitting and their implications for external validity are further discussed in the *Threat to Validity section.*

### Architecture of the SGA-DT framework

**Fig 1:**
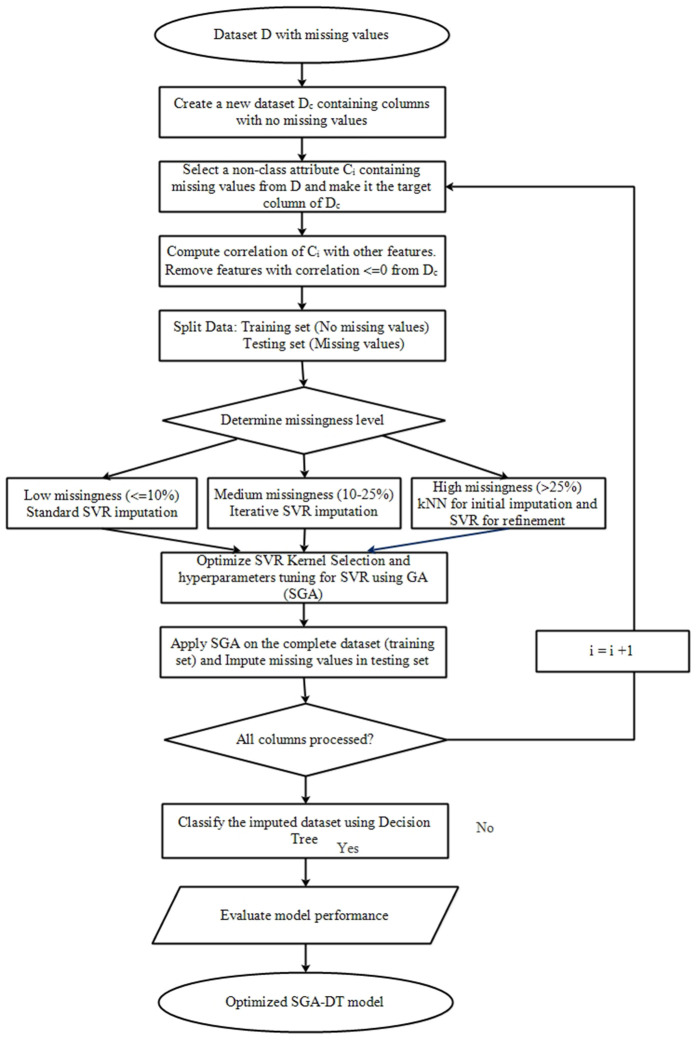
Proposed SGA-DT framework.

The proposed SGA-DT framework presents an adaptive and intelligent solution for handling missing values in non-class attributes while ensuring reliable classification. The framework operates in two distinct yet integrated phases: (1) Missing value imputation using GA-optimized SVR, and (2) Classification using DT. By dynamically selecting the imputation strategy based on the level of missingness and optimizing SVR parameters using GA, the SGA-DT framework tries to enhance both the quality of imputed data and the overall predictive performance. Fig 1 illustrates the schematic diagram of the proposed SGA-DT framework. The choice to integrate GA and DT into the SGA-DT framework is motivated by their complementary roles in optimization and interpretability. Since the quality of SVR-based imputation depends strongly on the choice of kernel and hyperparameters, GA is employed to perform joint, data-driven optimization of these parameters, enabling the SVR model to adapt to heterogeneous missingness patterns and attribute distributions. Moreover, as SVR is not inherently interpretable, Decision Tree classifier is incorporated after imputation to provide transparent, rule-based predictions, which is essential for healthcare decision-making. Thus, GA ensures optimal and stable imputations, while DT ensures interpretability and clinically meaningful classification outcomes.

### Missing value imputation using adaptive SVR

Before missing values can be imputed, the dataset *D* must be processed and refined to ensure a robust imputation strategy. Let the dataset *D* be defined as:


$$D=\{x_{i},y_{i}{\}}_{i=1}^{n}$$ (8)

where, $x_{i}\in {\mathbb{R}}^{p}$ represents the feature vector of instance *i*, *y*_*i*_ denotes the corresponding class label, and *p* is the total number of attributes in the dataset. Among these, *r* denotes non-class attributes and *q* denote class attributes, so that *p* = *r* + *q* describes the attribute partition used in the subsequent imputation procedure. To facilitate imputation, the dataset *D* containing missing values is taken. A new dataset *D*_*c*_ is constructed by retaining only the complete (non-missing) attributes, ensuring that reliable data is available for imputation. To facilitate the imputation process, a non-class attribute *C*_*i*_ with missing values is selected, which becomes the target column for imputation.

Although columns with no missing values are initially selected to form *D*_*c*_, this step does not eliminate informative attributes permanently. Instead, it isolates complete attributes to establish a stable reference space for correlation analysis and model training. Using incomplete columns during imputation can introduce bias and noise because the learning algorithm would rely on partially observed data with inconsistent variance. Therefore, *D*_*c*_ provides a clean basis for estimating correlations and fitting the SVR model, which subsequently imputes the removed (incomplete) columns one by one. This approach preserves all original attributes in the final dataset while ensuring each imputation is guided by statistically reliable predictors rather than partially missing ones. The dataset is then analyzed to compute the correlation between *C*_*i*_ and other features in *D*_*c*_. Features with correlation values ≤0 are removed to prevent irrelevant attributes from degrading SVR imputation quality [28].


$$\rho_{\left(C_{i},X_{j}\right)}=\frac{\sum (C_{i}-\bar{C_{i}})(X_{j}-\bar{X_{j}})}{\sqrt{\sum (C_{i}-\bar{C_{i}}{)}^{2}}\sqrt{\sum (X_{j}-\bar{X_{j}}{)}^{2}}}$$ (9)

where, $\rho_{\left(C_{i},X_{j}\right)}$ represents the Pearson correlation coefficient [29]. To effectively train the SVR model, the dataset is split into two subsets: Training set, containing instances where *C*_*i*_ has no missing values, and Testing set, which contains instances where *C*_*i*_ has missing values and will be used for imputation. This ensures that SVR learns from complete instances before predicting missing values. SVR is applied to predict the missing values of *C*_*i*_ based on the observed values in *D*_*c*_. The imputation process varies based on the level of missingness.

The missingness percentage of the selected attribute *C*_*i*_ is assessed to determine the appropriate imputation strategy. The missing value percentage in the datasets is determined by calculating the proportion of instances that contain at least one missing attribute. This metric provides an accurate representation of the extent of missing data by considering instances that are partially incomplete, rather than just the total number of missing entries. This adaptive strategy ensures that attributes with varying degrees of missingness are handled optimally. Based on the missingness percentage, the following strategies are applied:

- Low Missingness: In this case, the percentage of missing values is ≤10%. When the missing data is minimal, the patterns and relationships in the available dataset remain largely intact. Hence, standard SVR imputation is directly applied to estimate missing values based on the correlation of the target attribute with other fully observed attributes. This ensures a reliable imputation without introducing additional computational complexity.
  $$C_{i}=f(X_{c})+\epsilon$$ (10)
  where, *C*_*i*_ represents the missing non-class attribute to be imputed, *f*(*X*_*c*_) is the SVR prediction function that estimates *C*_*i*_ based on the observed attributes in the dataset *X*_*c*_, and ϵ is the residual error term, capturing small deviations due to noise in the data.
- Medium Missingness: In this case, the percentage of missing values lies between 10% and 25%. In such cases, a single pass of imputation may not be sufficient, as missing values might still affect model predictions. To improve accuracy, an iterative SVR imputation strategy is employed, where the initially imputed values are used as inputs for subsequent refinements. The process continues over multiple iterations until the imputed values converge to stable estimates, minimizing the impact of missing data.
  $$C_{i}^{\left(t+1\right)}=f(X_{c},C_{i}^{\left(t\right)})+\epsilon$$ (11)
  where, $C_{i}^{\left(t\right)}$ represents the imputed value at iteration *t*, and $C_{i}^{\left(t+1\right)}$ is the updated imputed value a*t* the next iteration. The function $f(X_{c},C_{i}^{\left(t\right)})$ uses both observed attributes *X*_*c*_ and the previous estimate of *C*_*i*_ to refine the imputation. The residual error term ϵ accounts for any small variations in prediction.
- High Missingness: When more than 25% of the values are missing, relying solely on SVR imputation may lead to unreliable predictions due to the lack of sufficient training data. In such cases, a two-stage hybrid imputation approach is applied:
  1. Initial KNN imputation: At first, KNN imputation is applied to provide a rough but structurally consistent estimate of missing values based on instance similarity. The imputed value is computed as the mean of the *k* nearest neighbors:
    $$C_{i}^{\left(0\right)}=\frac{1}{k}\sum_{j=1}^{k}C_{i,j}$$ (12)
    where $C_{i}^{\left(0\right)}$ is the initial imputed value for instance *i*, and *C*_*i,j*_ denotes the imputed value for attribute *C*_*j*_ of instance *i*, obtained from its *k* nearest neighbors. After identifying the *k* nearest neighbors with observed values for attribute *C*_*j*_, the imputed value for *C*_*i,j*_ is computed from this neighborhood. For numerical attributes, *C*_*i,j*_ is set to the mean of the *k* neighbors’ values in column *j*, whereas for categorical attributes, the most frequent category (majority vote) among the *k* neighbors is used. This ensures that the imputation criterion respects the data type while reflecting the local structure around instance *i*.
  2. Refinement using SVR: After the initial KNN imputation provides rough estimates of the missing values, SVR is applied as a second-stage refinement step. At this point, the dataset, now structurally complete, enables SVR to be effectively trained. By learning from the approximate but complete input space, SVR captures complex, non-linear relationships that KNN alone cannot model. This refinement process adjusts the initial estimates to better align with the underlying data distribution, resulting in more accurate and reliable imputations, especially under high missingness conditions.

In existing imputation studies, missingness levels are often categorized using broad empirical ranges, with values below roughly 5–10% considered low, 10–30% as moderate, and higher percentages as high. These ranges are used only to define experimental regimes rather than to dictate how individual imputation algorithms operate. Following this general practice, the present work selects $\tau_{1}=10\%$ and $\tau_{2}=25\%$ as specific cut-offs within these commonly used ranges to define low, medium, and high missingness regimes for our adaptive SGA-DT strategy.

### SVR optimization for improved imputation

To enhance the accuracy of missing value imputation, the SVR model requires careful tuning of its hyperparameters. In this work, GA is employed to optimize the key hyperparameters of SVR, ensuring that the imputed values closely align with the true data distribution. The crucial hyperparameters optimized using GA include:

- Kernel Function (*K*(*x*, *x*′)): It determines how input features are transformed into a higher-dimensional space for better regression accuracy.
- Regularization Parameter (*C*): It controls the trade-off between model complexity and training error [30].
- Epsilon-insensitive Loss (ϵ): It defines the margin of tolerance for regression predictions.
- Kernel Parameter (γ): It regulates the influence of training instances in the feature space.
- Polynomial Degree (*d*): This parameter is specific to the polynomial kernel. It defines the flexibility and complexity of the regression surface.

By optimizing these parameters, the proposed model ensures that the SVR model generalizes well to different datasets, leading to robust missing value imputation.

#### SVR kernel selection.

The kernel function plays a pivotal role in SVR by defining how input features are mapped into higher-dimensional spaces, enabling the model to learn complex relationships [31]. In the proposed SGA-DT framework, the kernel is not selected manually; instead, GA dynamically determines the most suitable kernel type-Linear, Radial Basis Function (RBF), or Polynomial based on the data characteristics and the relationship between the target attribute and its predictors. The following kernel functions are considered:

- Linear Kernel: It is used when the relationship between the target attribute and its correlated features is approximately linear. It is computationally efficient and suitable for datasets where linear transformations are sufficient to capture the underlying structure.
  $$K(X,X^{\prime })=X^{T}X^{\prime }$$ (13)
- Radial Basis Function (RBF) Kernel: The RBF kernel is applied when the data exhibits non-linear patterns. It maps the input into an infinite-dimensional space, allowing the SVR to capture complex, localized variations.
  $$K(X,X^{\prime })=\exp(-\gamma ||X-X^{\prime }|{|}^{2})$$ (14)
  where γ is the kernel coefficient that controls the spread of influence of training points.
- Polynomial Kernel: It is suitable for capturing moderately complex and global non-linear relationships. It introduces interactions of different degrees between features, making it more expressive than the linear kernel while retaining interpretability.
  $$K(X,X^{\prime })=(\gamma X^{T}X^{\prime }+r{)}^{d}$$ (15)
  where γ is the kernel coefficient, *r* is a free constant (typically set to 0 or 1), and *d* is the degree of the polynomial.

During optimization, GA simultaneously selects the best kernel and tunes its associated hyperparameters (*C*, ϵ, γ, and *d* for polynomial) to minimize the mean squared error on the validation set. This dynamic kernel selection ensures that the SVR model is well-adapted to the data structure, enabling more accurate and robust imputation.

#### Genetic algorithm for hyperparameter optimization:

To enhance the accuracy of missing value imputation, the SVR model is configured with the most suitable kernel and carefully tuned hyperparameters. In the proposed model, GA is employed to simultaneously select the optimal SVR kernel type such as Linear, RBF, or Polynomial and optimize its associated hyperparameters. These include the regularization parameter *C*, the epsilon-insensitive margin ϵ, and kernel-specific parameters such as the kernel coefficient γ (for RBF and Polynomial kernels) and the polynomial degree *d* (for the Polynomial kernel). This kernel-aware optimization strategy allows the SVR model to effectively capture the underlying data patterns, leading to robust and accurate imputation results. The detailed configuration of hyperparameters and genetic operators used in the proposed GA-based optimization is summarized in Table 3.

1. Initialize population: A population of candidate SVR configurations is randomly generated. Each configuration includes the kernel type and its associated hyperparameters:
  $$\text{Population}=\{({\text{kernel}}_{1},C_{1},\gamma_{1},\epsilon_{1},d_{1}),\;({\text{kernel}}_{2},C_{2},\gamma_{2},\epsilon_{2},d_{2}),\ldots ,\;({\text{kernel}}_{N},C_{N},\gamma_{N},\epsilon_{N},d_{N})\}$$ (16)
  Each individual in the population is encoded as a chromosome that includes:
  $${\text{Chromosome}}_{i}=({\text{kernel}}_{i},C_{i},\gamma_{i},\epsilon_{i},d_{i})$$
  Here, *kernel*_*i*_ is a categorical gene representing the SVR kernel type (Linear, RBF, or Polynomial), *C*_*i*_ is the regularization parameter sampled from a logarithmic scale, $\gamma_{i}$ is the kernel coefficient (used in RBF and Polynomial kernels), $\epsilon_{i}$ is the epsilon-insensitive margin, and *d*_*i*_ is the polynomial degree (used only for the Polynomial kernel). This representation enables the GA to simultaneously explore both kernel selection and kernel-aware hyperparameter optimization.
2. Evaluate fitness: Each chromosome in the population encodes a candidate SVR configuration, including the kernel type and its associated hyperparameters. During fitness evaluation, only the parameters relevant to the selected kernel are used to train the SVR model. The trained SVR model is used to impute missing values, and the quality of imputation is assessed using the MSE between the actual and predicted values of the non-class attribute:
  $$\text{MSE}=\frac{1}{N}\sum_{i=1}^{N}(C_{i}-{\hat{C}}_{i}{)}^{2}$$ (17)
  Here, *C*_*i*_ is the actual observed value and ${\hat{C}}_{i}$ is the value predicted by the SVR model. MSE serves as the fitness function in GA, guiding the selection of candidate solutions that produce more accurate imputations.
3. Selection: In this work, tournament selection method is applied to choose high-quality individuals from the current population. A small subset of chromosomes is randomly selected, and their fitness values (based on MSE) are compared. The chromosome with the lowest MSE indicating the best imputation performance, is chosen as a parent for the next generation. This process is repeated to build a mating pool for crossover and reproduction.
4. Crossover: Selected parents undergo crossover to produce offspring that inherit traits from both parents. In the proposed work, chromosomes encode both kernel type and associated SVR hyperparameters. A combination of uniform and blended crossover is used:
  - Kernel type: Categorical gene (Linear, RBF, Polynomial) — inherited using *uniform crossover*, i.e., randomly chosen from either parent.
  - Continuous Parameters: The real-valued parameters *C*, γ, and ϵ are combined using *blended crossover*:
    $$C_{\text{new}}=\alpha C_{1}+(1-\alpha )C_{2},\;\gamma_{\text{new}}=\alpha \gamma_{1}+(1-\alpha )\gamma_{2},\;\epsilon_{\text{new}}=\alpha \epsilon_{1}+(1-\alpha )\epsilon_{2}$$
  - Polynomial Degree: As it is integer-valued, it is handled using *randomized averaging or rounding*:
    $$d_{\text{new}}=\left\lfloor \alpha d_{1}+(1-\alpha )d_{2}+0.5\right\rfloor$$
  - Here, α∈[0,1] is a blending factor randomly chosen for each crossover event. This hybrid strategy allows for effective mixing of parent traits while respecting the nature of each gene (categorical, continuous, or discrete).
5. Mutation: To maintain genetic diversity and avoid premature convergence, mutation is applied by introducing small random changes to one or more genes in the chromosome. The mutation strategy is tailored to the type of gene:
  - Kernel Type (categorical): With a low probability, the kernel type is randomly switched to one of the other available options (Linear, RBF, Polynomial).
  - Continuous Parameters (C,γ,ϵ): A small noise term δ is added to each parameter to introduce variability and help explore new regions of the search space:
    $$x_{\text{new}}=x+\delta$$ (18)
    where x∈{C,γ,ϵ}, and δ is sampled from a uniform or Gaussian distribution.
  - Degree: For Polynomial kernel, the degree is adjusted by a small integer:
    $$d_{\text{new}}=d+\Delta ,\;\Delta \in \{-1,0,1\}$$ (19)
    The mutated degree is clipped to remain within a valid predefined range (e.g., 2–5).

**Table 3:** SVR hyperparameters and genetic algorithm configuration.

Parameter	Range / Values	Initialization	Mutation Strategy
Kernel Type	Linear, RBF, Polynomial	Random choice from set	Random re-selection
Regularization (*C*)	[0.1, 1000]	Log-uniform sampling	Re-sampling within [0.1, 1000]
Epsilon (ϵ)	[0.001, 1.0]	Uniform sampling	Re-sampling within [0.001, 1.0]
Kernel Coefficient (γ)	[$10^{-5}$, 10]	Log-uniform sampling	Re-sampling within [$10^{-5}$, 10]
Polynomial Degree (*d*)	2, 3, 4, 5	Random integer from set	±1, clipped to [2, 5]
Selection Method	Tournament Selection (size = 2)		
Crossover Type	Uniform (categorical), Blended (continuous)		
Crossover Probability	0.8		
Mutation Probability	0.1 per gene		

This mutation strategy supports kernel-aware exploration and ensures that each gene evolves in a manner appropriate to its data type.

The GA proceeds through multiple generations until the minimal fitness value (i.e., the lowest MSE) is obtained, indicating that no further improvement can be achieved. After the optimization process, the SVR configuration with the lowest MSE is selected, including the best-performing kernel type (Linear, RBF, or Polynomial) and its optimized hyperparameters. This kernel-aware GA-driven search ensures that the imputation model is both accurate and well-aligned with the underlying data structure. The selected SVR model is then applied to impute the missing values. Algorithm 1 outlines the step-by-step procedure of the proposed SGA-DT framework. Initially, the adaptive imputation strategy is presented (lines 12–20), where the choice of SVR variant (standard, iterative, or KNN + SVR) taken by considering the level of missingness. The GA-based optimization of the SVR model is presented in second part from lines 21–27, which includes kernel selection and tuning of hyperparameters (C, ϵ, γ and *d*). This optimization is applied only when SVR is used, ensuring the model is well adapted to the imputation task. The subsequent steps involve imputation followed by classification using DT, and then evaluating the model performance.

**Algorithm 1 GA-optimized SVR imputation followed by decision tree classification**



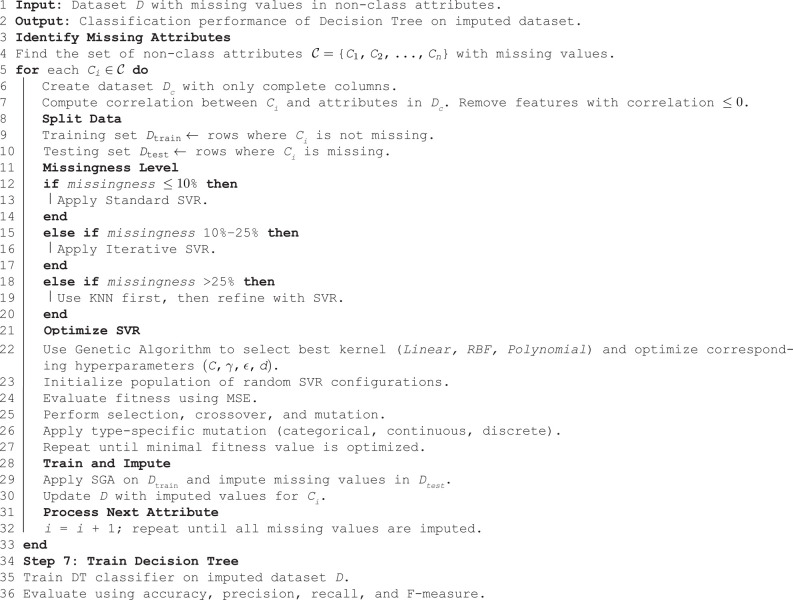



### Classification using DT

Once the missing values are imputed, the complete dataset is used to train the DT. The overall procedure for decision tree induction is illustrated in Algorithm 2, which builds the tree based on recursive partitioning of the data and selection of optimal split conditions. The algorithm begins with a training set consisting of instances and their corresponding class labels. Initially, an empty node *N* is created. If all instances belong to the same class, the node is labeled accordingly. In case the attribute list becomes empty, the node is labeled with the majority class, following the majority-voting rule [32]. Otherwise, the best attribute is selected for splitting based on attribute selection criteria. The subsequent steps guide the recursive splitting of data tuples based on attribute types. For discrete and binary attributes, splitting occurs based on distinct values, while for continuous attributes, midpoints between adjacent sorted values are considered. A critical component in this process is the splitting criterion, which identifies the attribute that best partitions the dataset into homogeneous subsets [33]. The partitioning continues until further splitting is not possible or meaningful, resulting in a fully induced tree [34].

**Algorithm 2 Decision tree induction**



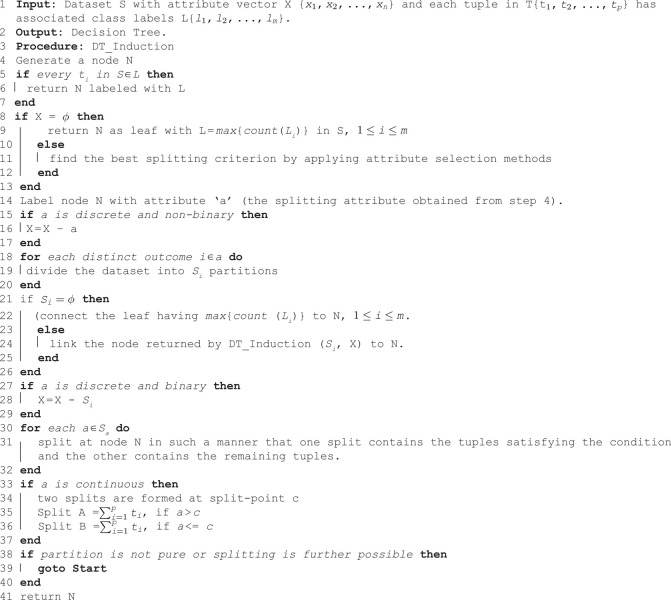



In our proposed SGA-DT framework, the classification and regression trees (CART) algorithm is used for classification [35]. CART constructs binary decision trees and can handle both discrete and continuous attributes. It employs the Gini Index (GI) as a splitting criterion, which measures node impurity and helps select the attribute that best separates the data. For a dataset *D*, the Gini Index is computed as [36]:


$$GI(D)=1-\sum_{i=1}^{n}P_{i}^{2},$$ (20)

where, $P_{i}=\frac{\left|S_{i}\right|}{\left|S\right|}$ is the ratio of number of tuples present in the dataset with respect to a particular class to the total number of tuples present in D. For a binary split with respect to an attribute ‘t’, GI can be calculated as:


$$GI_{t}(D)=\sum_{i=1}^{2}\frac{\left|D_{i}\right|}{\left|D\right|}GI(D_{i})$$ (21)

where, *D*_*i*_ is the gini index with respect to a partition. Due to the binary split on attribute t, the reduction in impurity is computed as:


$$GI_{red}(t)=GI(D)-GI_{t}(D)$$ (22)

All feasible binary splits are considered for each attribute, and the split yielding the maximum impurity reduction, with minimum *GI*_*red*_(*t*) is chosen. For continuous-valued attributes, CART evaluates all possible midpoints between adjacent values, similar to ID3 [37].

### Computational complexity analysis

The overall computational complexity of the SGA-DT framework for missing value prediction and classification can be evaluated by adding up the time complexities of each step in the algorithm. The computational complexity of the proposed SGA-DT imputation and classification algorithm is primarily influenced by three phases: missing value imputation using SVR, hyperparameter optimization using GA, and classification using DT. The first phase involves computing correlations between the target attribute and other available features, which takes *O*(*nm*) time, where *n* is the number of features and *m* is the number of samples. Splitting the dataset into training and testing subsets requires *O*(*m*) time, while training the SVR model incurs a complexity of *O*(*m*^3^) in the worst case due to matrix operations, which can be optimized to *O*(*m*^2^) with efficient kernel approximations. Predicting missing values for each instance takes O(m·d) time, where *d* is the number of missing values. The second phase, hyperparameter tuning using GA, optimizes SVR parameters (C,γ,ϵ) by iterating over a population of candidate solutions. Each generation requires evaluating *P* individuals, and since each SVR training takes *O*(*m*^2^), the per-generation complexity is $O(P\cdot m^{2})$, leading to an overall GA complexity of $O(G\cdot P\cdot m^{2})$ for *G* generations. Finally, the decision tree classifier is trained on the imputed dataset, with a training complexity of O(mlogm), assuming a balanced tree structure. The classification of test instances requires O(logm) operations, making the overall DT complexity O(mlogm). The dominant computational factor is the SVR training complexity (*O*(*m*^3^) in the worst case) and GA-based hyperparameter tuning ($O(G\cdot P\cdot m^{2})$), making the algorithm computationally intensive for large datasets. However, techniques such as kernel approximations for SVR, parallel GA execution, and decision tree pruning can significantly reduce computation time. Thus, while the SGA-DT framework offers high imputation accuracy and classification performance, its computational efficiency largely depends on dataset size, the number of features, and GA parameters, making it well-suited for medium to large-scale datasets with proper optimizations.

## Experimental results

The proposed SGA-DT framework has been implemented using Python and executed on a system with an Intel Core i7 processor, 8 GB RAM, and a 64-bit Windows operating system. The implementation utilizes libraries such as scikit-learn for SVR, decision tree classification, and evaluation metrics; DEAP for GA-based hyperparameter optimization; and NumPy, Pandas, and Matplotlib for data handling and visualization. All experiments were performed within the Jupyter Notebook environment for ease of testing and reproducibility.

### Datasets

To evaluate the effectiveness of the proposed SGA-DT framework, we considered a diverse collection of datasets obtained from well-established repositories including UCI, KEEL, and Kaggle. These datasets exhibit varying levels of missingness across numerical and categorical attributes, enabling a thorough assessment of the framework under different data quality conditions. The selected datasets span multiple domains such as healthcare, environmental science, with a particular focus on healthcare datasets to demonstrate the model’s applicability in critical decision-making contexts. To ensure generalizability, we also include two synthetic datasets with controlled missing value patterns generated under the missing at random (MAR) pattern. These datasets support systematic evaluation of the model’s performance on high-dimensional and mixed type data. Table 4 summarizes the characteristics of all datasets used, including the number of instances, attribute types, and missing value distributions. The proposed SGA-DT framework was trained and evaluated independently on each dataset without any merging or joint training. Moreover, missing-value imputation and GA-based SVR optimization were performed exclusively within the training folds of each dataset under 5-fold cross-validation. As no information from test folds or external datasets is used during imputation or optimization, this design avoids data leakage and reduces the risk of overfitting associated with amalgamated training.

- Breast Cancer [38]: The Breast Cancer dataset, sourced from the Oncology Institute and accessed via KEEL, is widely used in medical data mining research. It captures demographic and pathological factors associated with breast cancer recurrence. The dataset has been frequently cited in oncology-related machine learning studies due to its real-world origin and interpretability of features.
- Mammographic [39]: It is obtained via the KEEL repository and originally collected at the Institute of Radiology, University of Erlangen-Nuremberg, supports the classification of breast mass lesions using BI-RADS features. It is primarily used to predict malignancy and is valued for its diagnostic relevance in radiology and clinical decision support applications.
- Hepatitis [40]: It is a medical dataset used for predicting patient survival. It includes laboratory test results and clinical observations. The missing values are found in numerical attributes such as bilirubin and albumin levels. This dataset is particularly useful for assessing the effectiveness of imputation in healthcare-related classification tasks.
- Air Quality [41]: It is used for predicting pollutant concentration levels in the environment based on chemical sensor readings and meteorological data. Due to its real-world application in environmental monitoring and public health, the dataset is well-suited for assessing predictive modeling techniques.
- Adult [42]: The Adult dataset, derived from the 1994 U.S. Census, is used to predict whether an individual earns more than $50K annually. It includes demographic and occupational features, with missing values occurring in categorical attributes such as work class and occupation. Proper encoding technique is used for handling these missing values effectively.
- KDD Cup 1998 [43]: It is used for binary and multi-class classification, predicting whether a donation will be made in a direct marketing campaign based on demographic and behavioral data. Due to its real-world application in customer relationship management and fundraising, the dataset is valuable for evaluating predictive modeling techniques while ensuring data confidentiality.
- Synthetic Data 1: This dataset has been generated to simulate high missingness conditions (>25%). It contains only numerical attributes, making it ideal for evaluating the robustness of imputation techniques in highly sparse data environments. The dataset ensures that the model is tested under extreme missingness scenarios.
- Synthetic Data 2: This dataset is designed to mimic real-world structured data with a mix of categorical and numerical attributes. It has moderate missing values, making it useful for assessing the imputation efficiency when handling both categorical and numerical missing data. The dataset is included to ensure a comprehensive evaluation of the proposed model.

**Table 4:** Characteristics of datasets.

Dataset	No. of Instances	No. of Attributes	Attribute Type	Missing Attribute Types	Missing Value %	Source
**Breast Cancer**	286	10	Mixed	Categorical	3.15%	KEEL
**Mammographic**	961	6	Integer	Integer	13.63%	KEEL
**Hepatitis**	155	20	Mixed	Real	48.39%	KEEL
**Air Quality**	9358	15	Mixed	Real	21.25%	Kaggle
**Adult**	48842	15	Mixed	Categorical	7.41%	KEEL
**KDD Cup 1998**	191779	481	Mixed	Real	11.24%	UCI
**Synthetic Data 1**	5000	15	Numerical	Real	27.6%	Synthetic
**Synthetic Data 2**	15000	30	Mixed	Mixed	10%	Synthetic

Both synthetic datasets were generated programmatically to simulate controlled missingness patterns. First, fully observed records were created with the numbers of instances, feature types, and dimensionalities reported in Table 4. Then, missing values were injected using a MAR mechanism, in which the probability that an entry in attribute *C*_*j*_ was set to missing depended on the observed values of auxiliary attributes rather than on the unobserved value itself. The corresponding missingness probabilities were calibrated so that the overall missingness levels matched the desired targets (e.g., high missingness for Synthetic Data 1 and moderate missingness for Synthetic Data 2).

The Mammographic dataset does not contain raw mammography images; instead, it consists of structured, tabular BI-RADS based features derived from mammographic examinations. In contrast, the Breast Cancer dataset comprises clinical and pathological attributes related to disease prognosis and recurrence, rather than radiological descriptors. Thus, while both datasets address breast cancer–related prediction tasks, they differ in the clinical stage and information source they represent, radiology-derived assessment features versus patient-level clinical and pathological variables. The Adult and KDD Cup 1998 datasets contain large volumes of data, making them suitable for validating the scalability of the proposed framework. Most datasets contain mixed or numerical attributes, with a few involving only categorical features. To enable SVR-based imputation, categorical attributes with missing values are transformed using label encoding, which assigns unique integers to categorical values while preserving their distinct identities. For example, in the Adult dataset, categorical variables such as *Workclass*, *Occupation*, and *Native-country* are encoded before imputation.

### Results and discussions

To assess the effectiveness of the proposed SGA-DT framework, we conducted a comparative analysis against various integrated frameworks that combine different imputation techniques (drop, mean, mode, median, KNN, LR, NN) with DT classification. These include statistical imputation models (Mean-DT, Median-DT, Mode-DT), a traditional model using row deletion (Drop-DT), and machine learning-based imputation models (KNN-DT, LR-DT, NN-DT). We have also used SVR-DT as a comparison model, where canonical SVR (without GA optimization) is used for imputation. To ensure consistency, DT is used as the classifier across all models.

The performance of all integrated frameworks was assessed using four evaluation metrics: accuracy, precision, recall, and F-measure. As shown in Table 5, the proposed SGA-DT framework consistently outperforms all other methods across most datasets. It achieves the highest accuracy on all eight datasets, with particularly strong results in Synthetic Data 1 (94.1%) and Adult (96.6%). SVR-DT, which employs canonical SVR for imputation, ranks second in most datasets, validating the impact of genetic algorithm-based optimization in SGA-DT. Among machine learning-based integrated frameworks, NN-DT and LR-DT also perform well, showing competitive accuracy and stability across datasets. Among the statistical methods, Median-DT demonstrates the best performance overall, whereas Mean-DT outperforms in a few specific cases. Drop-DT consistently records the lowest accuracy due to information loss caused by discarding incomplete rows. The proposed SGA-DT framework consistently outperforms all other frameworks, achieving the highest average accuracy (92.77%), followed by SVR-DT (90.81%) and NN-DT (89.87%). In terms of precision, SGA-DT again leads with an average of 91.39%, while SVR-DT and NN-DT follow with 89.50% and 89.10%, respectively. For recall, SGA-DT records the top value of 91.38%, compared to SVR-DT (89.09%) and NN-DT (88.52%). Similarly, SGA-DT achieves the highest F-measure (91.48%), indicating a well-balanced trade-off between precision and recall.

**Table 5:** Performance results of various integrated frameworks across all the datasets.

Breast Cancer					Mammographic				
	Accuracy	Precision	Recall	F-measure		Accuracy	Precision	Recall	F-measure
**Drop-DT**	0.715	0.762	0.719	0.739	**Drop-DT**	0.618	0.643	0.678	0.66
**Mean-DT**	0.758	0.732	0.744	0.738	**Mean-DT**	0.702	0.671	0.698	0.684
**Mode-DT**	0.796	0.803	0.812	0.807	**Mode-DT**	0.723	0.678	0.711	0.694
**Median-DT**	0.802	0.831	0.819	0.825	**Median-DT**	0.755	0.718	0.692	0.705
**KNN-DT**	0.871	0.854	0.847	0.85	**KNN-DT**	0.823	0.811	0.782	0.796
**LR-DT**	0.868	0.849	0.858	0.853	**LR-DT**	0.768	0.734	0.791	0.761
**NN-DT**	0.892	0.878	0.867	0.872	**NN-DT**	0.814	0.799	0.819	0.808
**SVR-DT**	0.92	**0.918**	0.891	0.904	**SVR-DT**	0.843	0.822	0.864	0.842
**SGA-DT**	**0.924**	0.91	**0.919**	**0.914**	**SGA-DT**	**0.91**	**0.842**	**0.898**	**0.869**
**Hepatitis**					**Air Quality**				
	**Accuracy**	**Precision**	**Recall**	**F-measure**		**Accuracy**	**Precision**	**Recall**	**F-measure**
**Drop-DT**	0.753	0.692	0.749	0.719	**Drop-DT**	0.718	0.674	0.716	0.694
**Mean-DT**	0.813	0.729	0.77	0.748	**Mean-DT**	0.756	0.778	0.736	0.756
**Mode-DT**	0.777	0.783	0.791	0.787	**Mode-DT**	0.759	0.779	0.739	0.758
**Median-DT**	0.814	0.809	0.813	0.811	**Median-DT**	0.792	0.772	0.759	0.765
**KNN-DT**	0.882	0.853	0.834	0.843	**KNN-DT**	0.841	0.826	0.819	0.822
**LR-DT**	0.85	0.822	0.821	0.821	**LR-DT**	0.834	0.766	0.779	0.772
**NN-DT**	0.823	0.818	0.827	0.822	**NN-DT**	0.835	0.82	0.812	0.816
**SVR-DT**	0.904	0.871	**0.901**	0.886	**SVR-DT**	0.858	0.845	0.831	0.838
**SGA-DT**	**0.932**	**0.898**	**0.901**	**0.899**	**SGA-DT**	**0.899**	**0.875**	**0.866**	**0.87**
**Adult**					**KDD Cup 1998**				
	**Accuracy**	**Precision**	**Recall**	**F-measure**		**Accuracy**	**Precision**	**Recall**	**F-measure**
**Drop-DT**	0.721	0.763	0.723	0.742	**Drop-DT**	0.766	0.654	0.702	0.677
**Mean-DT**	0.746	0.792	0.782	0.787	**Mean-DT**	0.798	0.707	0.713	0.709
**Mode-DT**	0.854	0.812	0.782	0.797	**Mode-DT**	0.754	0.702	0.712	0.707
**Median-DT**	0.857	0.822	0.821	0.821	**Median-DT**	0.772	0.693	0.722	0.707
**KNN-DT**	0.907	0.899	0.89	0.894	**KNN-DT**	0.834	0.823	0.818	0.82
**LR-DT**	0.893	0.881	0.871	0.876	**LR-DT**	0.821	0.766	0.755	0.76
**NN-DT**	0.912	0.897	0.893	0.895	**NN-DT**	0.823	0.816	0.82	0.818
**SVR-DT**	0.953	0.925	**0.922**	**0.923**	**SVR-DT**	0.867	0.865	0.863	0.864
**SGA-DT**	**0.966**	**0.927**	0.919	0.922	**SGA-DT**	**0.913**	**0.922**	**0.93**	**0.926**
**Synthetic Data 1**					**Synthetic Data 2**				
	**Accuracy**	**Precision**	**Recall**	**F-measure**		**Accuracy**	**Precision**	**Recall**	**F-measure**
**Drop-DT**	0.682	0.643	0.717	0.678	**Drop-DT**	0.699	0.672	0.647	0.659
**Mean-DT**	0.809	0.702	0.737	0.719	**Mean-DT**	0.786	0.715	0.735	0.725
**Mode-DT**	0.776	0.706	0.743	0.724	**Mode-DT**	0.779	0.754	0.739	0.746
**Median-DT**	0.776	0.749	0.749	0.749	**Median-DT**	0.82	0.729	0.743	0.736
**KNN-DT**	0.899	0.871	0.878	0.874	**KNN-DT**	0.91	0.872	0.874	0.873
**LR-DT**	0.82	0.791	0.795	0.793	**LR-DT**	0.897	0.886	0.896	0.89
**NN-DT**	0.879	0.867	0.828	0.847	**NN-DT**	0.912	0.892	0.891	0.891
**SVR-DT**	0.895	0.865	0.855	0.859	**SVR-DT**	0.923	0.915	0.924	0.919
**SGA-DT**	**0.941**	**0.929**	**0.918**	**0.923**	**SGA-DT**	**0.95**	**0.945**	**0.936**	**0.940**

**Fig 2:**
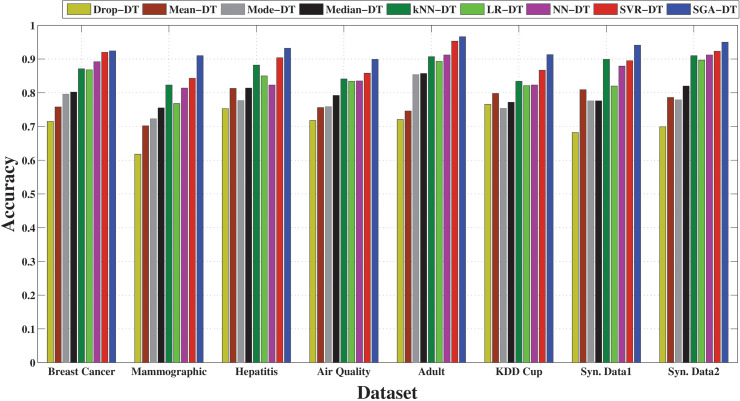
Performance comparison of SGA-DT with other integrated frameworks in terms of accuracy.

Fig 2 illustrates the comparative performance of all integrated frameworks in terms of accuracy across eight datasets. Drop-DT consistently records the lowest accuracy. Among statistical frameworks, Median-DT outperforms Mean-DT and Mode-DT in most cases, demonstrating its robustness against outliers. The machine learning-based frameworks significantly outperform statistical frameworks, with SGA-DT achieving the highest accuracy across all datasets. Notably, in datasets like Hepatitis, Adult, and Breast Cancer, SGA-DT achieves an accuracy exceeding 92%, highlighting the benefit of GA optimization. SVR-DT also performs consistently well, often ranking second, followed closely by NN-DT. LR-DT and KNN-DT show moderate accuracy but trail SGA-DT by a noticeable margin.

**Fig 3:**
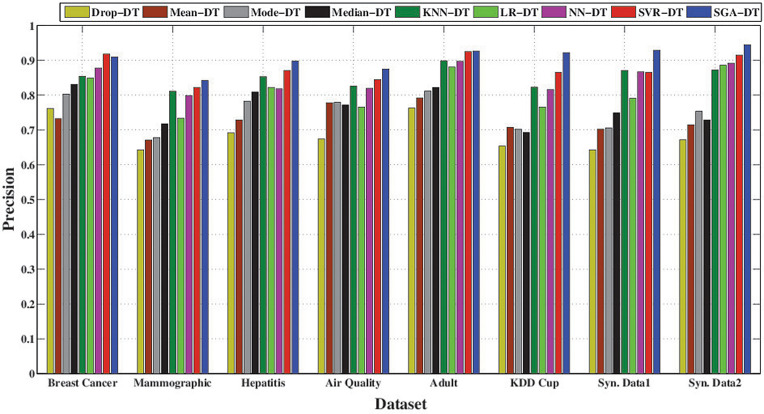
Performance comparison of SGA-DT with other integrated frameworks in terms of precision.

The precision-based comparison in Fig 3 highlights the superior performance of the SGA-DT framework across all datasets. It achieves the highest precision in every case, with particularly strong results in Synthetic Data 1 (92.9%), Adult (92.7%), and Synthetic Data 2 (94.5%). SVR-DT emerges as the second-best model overall, with close precision values in several datasets, for example, 91.8% in Breast Cancer and 92.5% in Adult. NN-DT also maintains good precision performance, with scores exceeding 86% across most datasets, followed by KNN-DT. Among the statistical frameworks, Median-DT exhibits relatively higher precision compared to Mean-DT and Mode-DT, although Mode-DT outperforms in certain instances. Drop-DT consistently shows the lowest precision, with particularly poor performance on the KDD Cup and Mammographic datasets, where its results fall significantly behind the other methods. The results confirm that machine learning-based imputation, particularly when enhanced with optimization (SGA-DT), preserves class relevant feature structure better than simple averaging or deletion methods, leading to fewer false positives and improved classification confidence.

**Fig 4:**
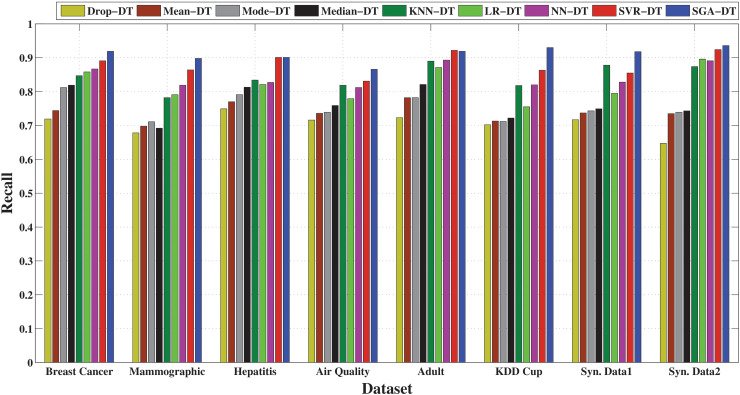
Performance comparison of SGA-DT with other integrated frameworks in terms of recall.

Fig 4 highlights the effectiveness of different imputation techniques in correctly identifying positive instances. The proposed SGA-DT consistently achieves the highest recall, with 93.0% in KDD Cup, 86.7% in Air Quality, 92.0% in Breast Cancer datasets. SVR-DT ranks second in most cases, demonstrating strong performance, followed closely by NN-DT and LR-DT. KNN-DT performs reliably, especially in the Adult and Synthetic Data 1 datasets. Among the statistical frameworks, Median-DT consistently outperforms Mean-DT and Mode-DT, whereas there is a deviation in case of Mammographic dataset. Drop-DT continues to show the lowest recall across all datasets, reaffirming that simply removing missing values adversely affects model sensitivity.

**Fig 5:**
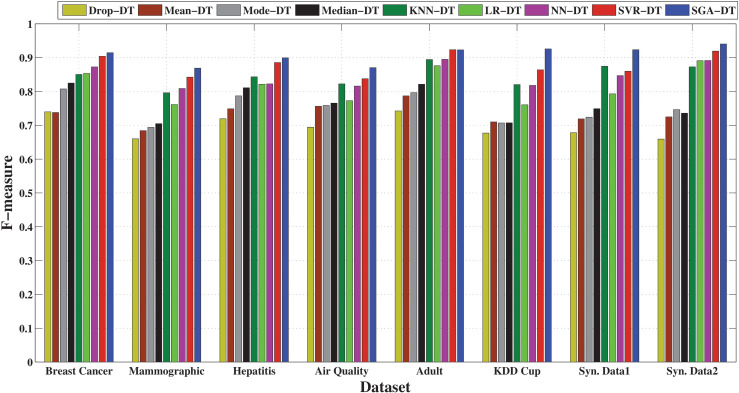
Performance comparison of SGA-DT with other integrated frameworks in terms of F-measure.

The F-measure comparison, illustrated in Fig 5, highlights the balance between precision and recall across different imputation strategies. SGA-DT consistently achieves the highest F-measure scores, with 91.4% in Breast Cancer, 92.3% in Adult, and 92.3% in Synthetic Data 1, confirming its effectiveness in maintaining classification reliability. SVR-DT follows closely in most cases maintaining a strong balance between precision and recall. KNN-DT also demonstrates consistent performance, achieving over 87% F-measure in most datasets and ranking among the top frameworks in several cases. Among statistical methods, Median-DT outperforms Mean-DT and Mode-DT across most datasets, suggesting greater stability in handling missing data. Drop-DT lags behind all other frameworks, with the lowest F-measure in nearly every dataset.

**Fig 6:**
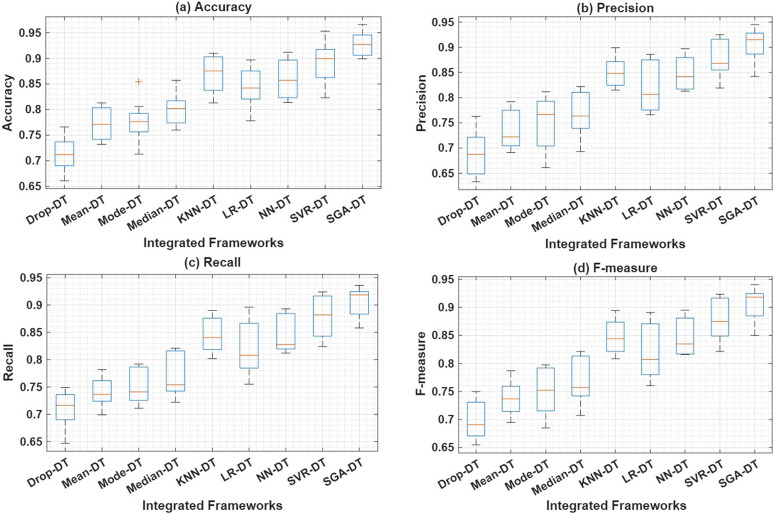
Boxplot analysis of performance metrics for SGA-DT and other integrated frameworks.

To make a comparative analysis of overall performance of all the integrated frameworks, box plot analysis is performed, which is shown in Fig 6. The analysis is performed based on each of the evaluation parameters by considering all the datasets. The results for each of the performance parameters are obtained by performing a five-fold cross-validation for each of the integrated frameworks on each of the considered datasets. The results are then shown in the form of a box plot. The box plot represents the maximum, minimum, average, 1/4th quartile, and 3/4th quartile values of each performance parameter.

Fig 6a presents the boxplot analysis for accuracy, while Fig 6b–6d illustrate the performance distributions for precision, recall, and F-measure, respectively. Across all four metrics, SGA-DT consistently outperforms other frameworks, achieving the highest median accuracy of approximately 92.7%, with values ranging from 89.9% to 96.6%. This narrow and elevated span reflects the robustness and reliability of the proposed model across diverse datasets. In comparison, other machine learning-based frameworks such as NN-DT and LR-DT exhibit competitive yet more variable accuracy distributions. NN-DT shows relatively high maximum accuracy but slightly lower median and wider interquartile range, indicating less consistency. LR-DT demonstrates moderate accuracy, but lower upper-bound accuracy. The KNN-DT framework performs better than LR-DT in terms of median accuracy.

Fig 6b presents the precision distribution across all integrated frameworks. SGA-DT achieves the highest and most stable precision across datasets, followed by SVR-DT. For precision, SGA-DT maintains a high median of 91.5%, with values ranging from 84.2% to 94.5%, indicating both high predictive quality and consistent performance in identifying true positives. Similarly, Fig 6b shows that SGA-DT achieves the highest median precision of 91.5%, followed by SVR-DT (86.8%) and KNN-DT (84.8%), highlighting its superior ability to minimize false positives. Among the statistical frameworks, Median-DT performs better than Mean-DT and Mode-DT, while Drop-DT records the lowest precision.

Fig 6c further illustrates that SGA-DT also maintains the highest recall (91.8%), confirming its reliability in capturing true positives. SVR-DT ranks second (88.2%), followed by NN-DT and KNN-DT, with LR-DT slightly behind. Statistical frameworks, particularly Mean-DT, show relatively consistent but lower recall values compared to the machine learning-based approaches. Fig 6d shows that SGA-DT achieves the highest F-measure (91.8%), confirming a well-balanced precision-recall trade-off. SVR-DT and KNN-DT follow with 87.5% and 84.4%, respectively. Among the statistical frameworks, Median-DT records the best F-measure, while Drop-DT consistently performs the worst across all metrics.

**Fig 7:**
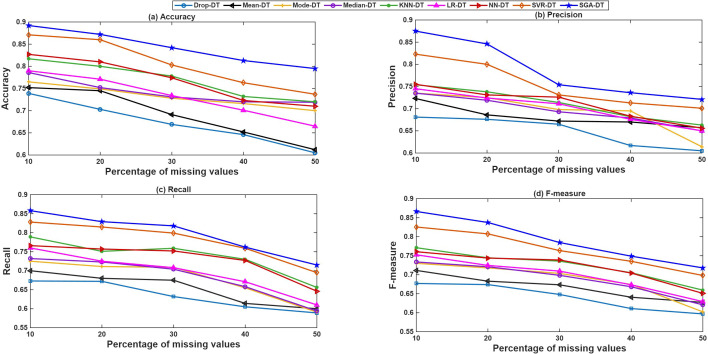
Effect of percentage of missing value on various performance parameters.

In addition to the experiments discussed earlier, an additional analysis was performed to evaluate how different levels of missingness affect model performance. For this purpose, missing values were introduced into the previously complete datasets using MAR mechanism, with missingness levels ranging from 10% to 50%. The resulting trends in accuracy, precision, recall, and F-measure for each missingness level are shown in Fig 7a–7d respectively. As expected, increasing missingness degrades the performance of all frameworks. However, SGA-DT consistently outperforms all other frameworks across every metric and missing value percentage. Notably, it maintains a clear advantage over SVR-DT, validating the effectiveness of genetic algorithm-based hyperparameter optimization and kernel selection. In Fig 7a, NN-DT, LR-DT, and KNN-DT display a linear drop in accuracy with increasing missing values. SVR-DT achieves better accuracy than these frameworks across most levels but is consistently outperformed by SGA-DT. SGA-DT achieves the highest accuracy at every point, demonstrating robust adaptation to incomplete data. Drop-DT shows the poorest performance, except at 50%, where it converges with Mean-DT.

Fig 7b shows that SGA-DT leads all frameworks in precision across all levels of missingness. SVR-DT performs well, ranking just below SGA-DT, which highlights the positive effect of GA optimization. At low missingness (10%), NN-DT edges slightly ahead of LR-DT, but their precision values converge at higher missing percentages. The recall analysis in Fig 7c reinforces this trend. SGA-DT achieves the highest recall values across the board, followed closely by SVR-DT. The performance gap between SGA-DT and SVR-DT grows with higher missingness, indicating that the adaptive imputation strategies in SGA-DT become more impactful as data quality decreases. Fig 7d confirms that SGA-DT maintains the highest F-measure at all missingness levels, demonstrating an effective balance between precision and recall. SVR-DT ranks second across most levels, again affirming the value of integrating GA into the imputation process. NN-DT and LR-DT perform comparably, slightly ahead of KNN-DT. At 50% missingness, all three converge, while Drop-DT consistently yields the lowest F-measure.

**Fig 8:**
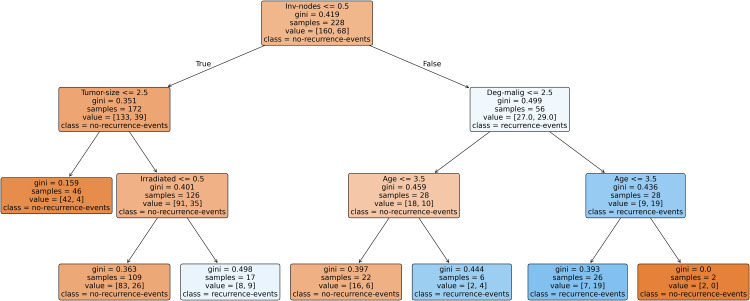
Pruned decision tree generated for Breast Cancer dataset (ccp_alpha = 0.003).

**Fig 9:**
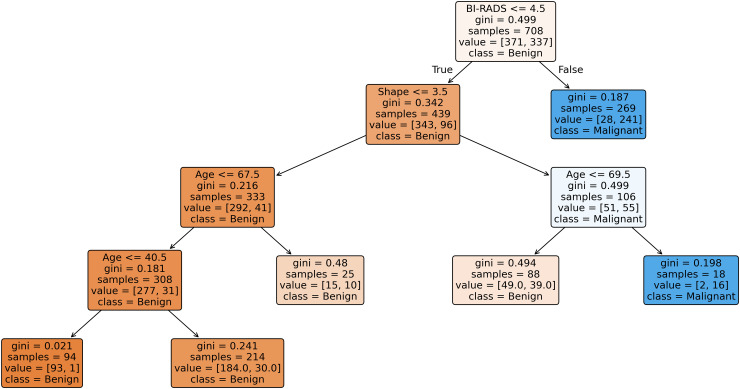
Pruned decision tree generated for Mammographic dataset (ccp_alpha = 0.003).

**Fig 10:**
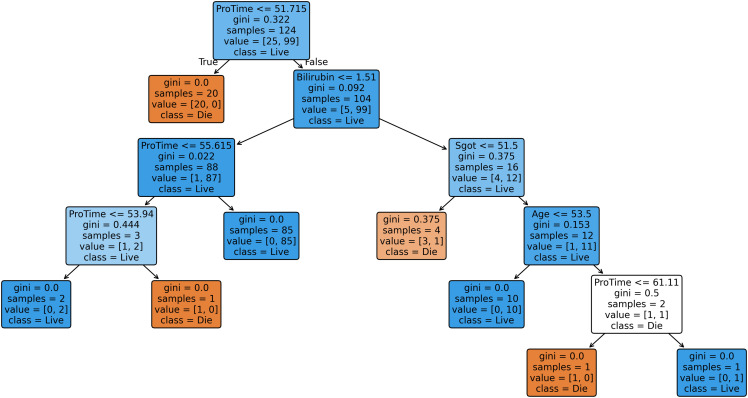
Pruned decision tree generated for Hepatitis dataset (ccp_alpha = 0.007).

In addition to the quantitative evaluation, model interpretability is further explored through pruned decision trees generated from selected imputed datasets. Since generating trees for all datasets would be space-intensive, three representative datasets, each corresponding to a distinct level of missingness (low, medium, and high) are chosen to illustrate the interpretability of the SGA-DT framework. Pruned decision trees are used instead of fully grown ones, as the later often produce cluttered, overlapping structures that hinder interpretability. To enhance generalization and simplify the tree structure, cost-complexity pruning (CCP) is applied using Scikit-learn, where the (ccp_alpha parameter balances accuracy and model simplicity. Fig 8 shows the pruned decision tree for the Breast Cancer dataset (low missingness: 3.15%). Similarly, Fig 9 represents the Mammographic dataset (medium missingness: 13.61%), and Fig 10 illustrates the Hepatitis dataset (high missingness: 48.39%).

Fig 8 illustrates the pruned decision tree generated for the Breast Cancer dataset. The tree begins with the most informative attribute, *Inv-nodes*, as the root node, followed by key predictors such as *Tumor-size*, *Irradiated*, and *Deg-malig*. The tree structure reflects clinically interpretable rules for predicting the likelihood of recurrence events. For example, patients with fewer involved nodes (*Inv-nodes* ≤ 0.5) and smaller tumor size are predominantly classified as *no-recurrence-events*. The model’s decisions align well with domain knowledge, and the pruning process (with (ccp_alpha = 0.003) effectively balances complexity and generalization by reducing overfitting while preserving interpretability. The use of *Age* and *Irradiated* attributes in lower branches further emphasizes their contribution to classification in more ambiguous cases.

Fig 9 illustrates the structure of the pruned decision tree for Mammographic dataset with ccp_alpha = 0.003. The tree was constructed to classify mammographic masses as benign or malignant based on five non-class attributes: BI-RADS assessment, Age, Shape, Margin, and Density. The pruning strategy effectively reduces model complexity while retaining critical decision boundaries. At the root node, the BI-RADS assessment emerges as the most discriminative feature, separating the dataset based on radiological severity. The left sub-tree captures benign cases through further splits on Shape and Age, achieving high purity (e.g., gini = 0.021 with 93 benign and 1 malignant case). On the right branch, malignant classifications are refined through Age-based splits, including a leaf with gini = 0.198, capturing 16 malignant and only 2 benign instances.

Similarly, Fig 10 shows the pruned decision tree generated for the Hepatitis datasets. In the Hepatitis tree (ccp_alpha = 0.007), ProTime and Bilirubin serve as the most influential features in predicting patient survival, with pure decision paths capturing clear distinctions between live and die classes. These decision trees not only contribute to accurate classification but also enhance the interpretability of the proposed SGA-DT framework, offering transparent, rule-based reasoning behind each prediction, thereby aligning with the adaptive and interpretable learning objectives of the framework.

## Ablation study

The contributions of the individual components within the proposed SGA-DT framework namely, SVR for imputation, GA-based hyperparameter optimization, and DT classification are analyzed through a detailed ablation study. While DT serves as the core classification framework in all model variants and remains constant throughout the study, the objective was to assess how the imputation strategy, particularly the inclusion of GA optimization in SVR affects the classification performance of DT. By keeping DT fixed and varying the imputation approach (Drop, canonical SVR, and GA-optimized SVR), we were able to evaluate the direct impact of the GA component in enhancing the effectiveness of the overall SGA-DT framework. Three model variants were compared in this ablation study:

- Drop-DT: A baseline model where instances with missing values are removed before classification using DT.
- SVR-DT: A variant employing canonical SVR for imputing missing values without GA optimization, followed by DT classification.
- SGA-DT (Proposed): Our complete model integrating GA-optimized SVR for imputation with DT as the final classifier.

Across all datasets and metrics, SGA-DT consistently outperforms both the Drop-DT and SVR-DT models. The improvement over Drop-DT is significant, highlighting that removing incomplete samples results in substantial information loss. While SVR-DT performs competitively and often ranks second, the consistent superiority of SGA-DT highlights the critical role of GA optimization in enhancing imputation quality. Specifically, SGA-DT achieves an average accuracy of 92.77%, exceeding that of SVR-DT (90.81%) and Drop-DT (81.29%).

This performance gain is further reflected in other metrics. SGA-DT achieves an average precision of 91.39%, recall of 91.38%, and F-measure of 91.48%, surpassing all alternative variants. The improvements are particularly evident in datasets with high dimensionality or complex missingness patterns, such as Adult and Synthetic Data 1, where optimized hyperparameters lead to better feature reconstruction and classification. This ablation confirms that the GA component significantly strengthens the imputation stage of the SGA-DT framework. By fine-tuning SVR parameters in a data-driven manner, GA enhances the preservation of class relevant patterns in imputed data, directly contributing to the superior classification performance as observed from the result.

## Threat to validity

While the SGA-DT framework offers a robust solution for missing value imputation and classification, several factors need careful consideration to ensure consistent performance across diverse datasets. Key aspects that may affect generalizability and possible refinements are discussed below:

- Although the proposed framework employs cost-complexity pruning to reduce overfitting in decision trees, challenges may still persist in high-dimensional or noisy datasets. Additional strategies such as regularization or ensemble methods like Random Forests can further enhance generalization.
- Although SVR-based imputation improves the reliability of the dataset, its effectiveness can diminish in the presence of extreme noise or very high missingness. While the framework utilizes KNN for initial imputation in such cases, incorporating robust preprocessing or outlier detection techniques may further enhance stability.
- GA-optimized SVR combined with DT enhances both imputation and classification accuracy. While this integrated approach adds moderate computational overhead for large datasets, the performance gains justify the cost. Future work may explore alternatives like Bayesian optimization to improve efficiency without compromising accuracy.
- The effectiveness of SVR heavily depends on the choice of kernel and its associated hyperparameters. An inappropriate kernel selection can reduce imputation quality. While GA-based selection is currently employed, incorporating advanced adaptive kernel learning methods could further enhance flexibility and model generalizability.
- Although the SGA-DT model adapts to different levels of missingness, its performance may degrade when missing values exceed 50% of the dataset. Nonetheless, the multi-strategy imputation mechanism (SVR, iterative SVR, and KNN-SVR refinement) provides a solid foundation. Future work could explore deep learning-based imputation approaches to better handle extreme missingness scenarios.

## Conclusion and future work

In this work, we presented SGA-DT, an adaptive and interpretable learning framework that integrates genetically optimized support vector regression with a decision tree for interpretable classification. The proposed model adaptively selects among three imputation strategies, standard SVR, iterative SVR, and KNN followed by SVR refinement, based on the percentage of missingness in non-class attributes. Genetic Algorithm is employed to jointly optimize the SVR kernel type and its hyperparameters, ensuring high-quality imputations across diverse data patterns. The imputed dataset is classified using decision tree, ensuring transparency through interpretable, rule-based outputs. The inclusion of cost-complexity pruning further supports model simplicity and trustworthiness, essential in healthcare scenario. Extensive evaluation across diverse datasets confirms that SGA-DT consistently outperforms other integrated frameworks, demonstrating strong adaptability and generalizability, particularly in healthcare prediction tasks where both accuracy and interpretability are crucial.

While SGA-DT shows promising results, several enhancements can further improve its capabilities. Future research could explore alternative regressors such as polynomial or Lasso regression, or deep learning-based models to handle complex nonlinear patterns in imputation. For classification, ensemble methods like Random Forest or XGBoost may improve robustness. Replacing GA with Bayesian optimization could reduce computational overhead, particularly for large-scale data. In extreme missingness scenarios (>50%), generative models such as GAN-based imputation may improve data reconstruction. Additionally, integrating explainable AI techniques and robust feature selection prior to imputation could further enhance the transparency and trustworthiness of the model.
